# A Salient and Task-Irrelevant Collinear Structure Hurts Visual Search

**DOI:** 10.1371/journal.pone.0124190

**Published:** 2015-04-24

**Authors:** Chia-huei Tseng, Li Jingling

**Affiliations:** 1 Department of Psychology, The University of Hong Kong, Hong Kong; 2 Graduate Institute of Neural and Cognitive Sciences, China Medical University, Taichung, Taiwan; Centre de Neuroscience Cognitive, FRANCE

## Abstract

Salient distractors draw our attention spontaneously, even when we intentionally want to ignore them. When this occurs, the real targets close to or overlapping with the distractors benefit from attention capture and thus are detected and discriminated more quickly. However, a puzzling opposite effect was observed in a search display with a column of vertical collinear bars presented as a task-irrelevant distractor [6]. In this case, it was harder to discriminate the targets overlapping with the salient distractor. Here we examined whether this effect originated from factors known to modulate attentional capture: (a) low probability—the probability occurrence of target location at the collinear column was much less (14%) than the rest of the display (86%), and observers might strategically direct their attention away from the collinear distractor; (b) attentional control setting—the distractor and target task interfered with each other because they shared the same continuity set in attentional task; and/or (c) lack of time to establish the optional strategy. We tested these hypotheses by (a) increasing to 60% the trials in which targets overlapped with the same collinear distractor columns, (b) replacing the target task to be connectivity-irrelevant (i.e., luminance discrimination), and (c) having our observers practice the same search task for 10 days. Our results speak against all these hypotheses and lead us to conclude that a collinear distractor impairs search at a level that is unaffected by probabilistic information, attentional setting, and learning.

## Introduction

Visual search is an integral part of our daily lives, and selective attention is the central mechanism that determines what relevant information is to be processed in our search behavior. In some cases, selective attention seems effortless and largely driven by stimuli properties [1], for example, when one area or object possesses a color or shape distinct from the rest, it catches attention immediately, even when these distinct features are of no help in finding our target. This effect is revealed when the searched-for target (e.g., a letter or oriented bar) is detected faster when it occurs on the area marked by task-irrelevant but salient features, such as color, shape, or abrupt onset [2–5]. This speed facilitation from a non-target (yet salient) object—termed “attentional capture”—suggests that attention is oriented by the stimuli properties.

**Fig 1:**
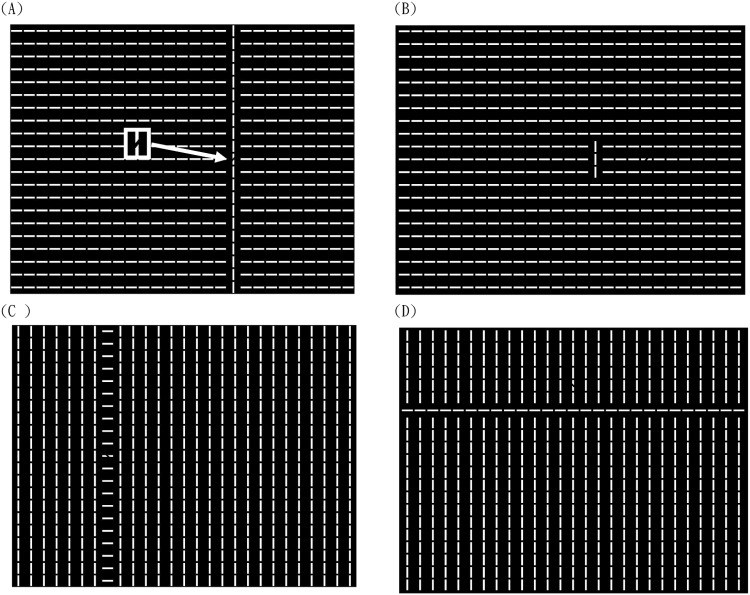
Examples of the search display in Jingling and Tseng (2013). The target is either overlapping (A or C) or non-overlapping (B or D) with the distractor. The distractor can be of long (21 bars) (A, C or D) or short collinear (3 bars) (B) in the distractor column. The configuration can be collinear vertical (A, B) or non-collinear (C), or collinear horizontal (D). The target is highlighted in (A) but not shown in experiment.

**Fig 2:**
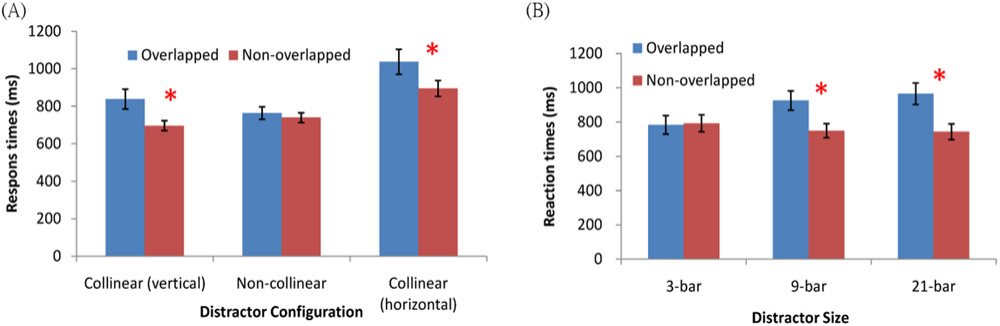
Illustration of main findings in Jingling and Tseng (2013). (A) The effect of distractor configuration. (B) The effect of distractor size.

Interestingly, Jingling and Tseng [6] reported an unexpected effect in a search display that contained a column of collinear bars that distinctly stood out from the search display (Fig 1). This column was a salient but task-irrelevant distractor, as it did not provide any location information to help target orientation discrimination (main task). Unexpectedly, when the target spatially overlapped with the distractor column (Fig 1A and 1C), observers’ search time increased—opposite to a reduction, as would be predicted from previous attention capture literature [2, 4]. Further, this search impairment is limited only when the distractor column is formed of snake-like collinear vertical bars (Fig 1A and 1D). When non-collinear (horizontal) bars were grouped to form the ladder-like distractor column (Fig 1C), the target overlapping with it received neither impairment (as in the collinear distractor condition) nor search facilitation (as predicted by attention capture theories) (Fig 2A). In both cases, overlapping targets contained identical local orientation contrast (both neighbored by two orthogonal and two parallel bars), so this ruled out a difference constituted by the salience computation based on local feature contrast. An additional experiment with collinear structure in the horizontal direction (Fig 1D) further suggested that collinear grouping of the global structure, instead of orientation of individual bar, was the critical factor driving the key difference (Fig 2A). We also found this search impairment was eliminated when the distractor column size was reduced (Figs 1B and 2B). As the local featural contrast (i.e., bottom-up defined salience) of the target was identical regardless of the distractor column length, our result strongly suggested that the collinear grouping determined the search interference. To summarize, our report highlights that the role of perceptual grouping on visual search, especially the principle of continuity or collinearity, is more important than previously thought. Nonetheless, how the perceptually grouped regions interact with the salience map and affect attentional capture are still open questions.

The view that attentional capture is highly automatic was challenged recently by several studies. For example, reduction of the probability of a color or onset singleton distractor in a search display lowered observers’ manual and eye movement response to target search [7–9], which implies statistical information might penetrate the salience computation derived from featural contrast and interfere with subsequent search. Besides, task-related demand such as attentional control setting is also reported to alter attentional capture. Folk and his colleagues [10, 11] found that salient task-irrelevant items could draw attention only when their features were contingent on top-down control settings, not when there was a mismatch between stimulus properties and what the observer was looking for. The requirement of match between task requirement and stimuli suggests the involvement of voluntary attention. Learning in visual search task was also reported to effectively inhibit attentional capture. Kelley and Yantis [12] reported that observers’ ability to suppress a task-irrelevant distracting onset improved after practice, and the learning effect was transferrable to other conditions. The learning generalization implies that the practiced suppression to be distracted was not a local adaptation effect, but more likely, a reflection of enhanced higher-level selection efficiency. The regulations from probability information, attentional control setting, and learning on attentional capture all argue against the view that attentional capture is driven automatically only by stimulus salience [13–17].

Before further efforts are invested into examination of the role of collinear grouping in selective attention, it is of great importance to examine whether the above-mentioned factors—namely, statistical information, attentional control setting, and learning—can be alternative accounts that lead to collinear search impairment. In Jingling and Tseng [6], we intended to make the collinear structure uninformative about the target location by equating the chance that a target overlaps on this collinear column and the other six possible columns (i.e., each at one out of seven, ~14%). It is possible that observers learned from the statistical regularity, intentionally suppressing the collinear distractor area due to its relatively low probability to coincide with target location compared with the rest of the areas in the search display (low-probability hypothesis). Several studies have demonstrated that observers can detect statistical regularities in the environment both with and without awareness [18–23, 36]. Eye movement studies also showed that people directed their eyes and attention faster to highly probable locations than to low-probability locations, without employing an explicit strategy to do so [24, 25]. If statistical regularity modulates search impairment, collinear or continuous grouping may not be the primary consideration.

Another possible source is attentional control setting, the mental set to look for helpful features to complete a task. Folk et al. [10] asked the participants to discriminate between a red T and a red L surrounded by white distractors. A non-informative cue of target location was shown before each target display. Usually this non-informative cue would not benefit target discrimination, but if the cue were red, which was congruent with the defining feature of the target, the observers’ response time was shortened. Subsequent studies found that such attentional control setting could be triggered by a distractor when the distractor shared a specific feature level with the target [10, 26] or in a different feature level but in the same feature dimension [10–11], when the distractor and the target were both the oddest ones [27], or when the distractor matched the task-wide expectation of how the target would appear [28–29]. In our case, the observers’ task was to discriminate between the orientation of a small gap, which indicated discontinuity between items, and the formation of a collinear distractor, which indicated continuity between items; thus, these two were in conflict in the attentional control setting, which might result in interference for the target task. If this is true, we would expect to reduce or remove the search impairment with another target task that did not require breaking the continuous distractor.

Finally, the collinear-selective impairment may be overcome by observers’ practice or learning to develop an optimal search strategy. In Jingling and Tseng [6], observers performed 10–12 of practice trials before completing a block of 400 trials, among which only 1/7 trials (~70 trials) contained targets overlapped with the collinear structure. Studies indicated that practice improved participants’ search ability, and in some cases, an initially inefficient (serial) task can become to an efficient (parallel) search task after hundred trials of learning [30–32]. This change was attributed to high-level strategic development because this effect can be easily generalized to a different location, eye, or task [30–32]. It is possible that our naïve observers were hampered in the overlapping target condition due to insufficient practice to optimize their search strategy. It is therefore desirable to further examine whether we can remove this search disadvantage in an overlapping condition after substantial practice.

In this study, we designed three experiments to further examine these alternative accounts that are not related to stimuli property: statistical learning, attentional control setting, and visual search learning.

## Experiment Design

Our study was approved by IRB at the University of Hong Kong and China Medical University. All participants provide their written informed consent approved by IRB prior to the study.

We first replicated our previous findings in Experiment 1. Then we increased the likelihood so that targets were six times (60%) more likely to appear on the task-irrelevant column than other possible column locations (four possible locations at 10% each) in Experiment 2. If the probability manipulation reduced or reversed the search impairment from the collinear distractor, it would support the view that statistic information may be the source of the damage (low-probability hypothesis). We found the search impairment persisted, which did not support this hypothesis.

In Experiment 3, we let the target be a brighter or darker bar relative to other elements, and the observers’ task was to discriminate the target’s luminance. Luminance alternation of grouped elements does not affect grouping strength of the global structure [33]; thus, a brighter or a darker target should not interfere with continuity of the collinear distractor. We still observed the same search impairment.

In Experiment 4, the observers underwent 10 days of search training. If the observers’ search strategy for a particular display was the bottleneck, we should expect to see the difference between overlapping and non-overlapping conditions gradually disappear through practice. We did not observe this trend, and the persistent collinearity-dependent impairment led us to reject the search strategy hypothesis.

## Experiment 1: Chance Level Overlapping Target

In this experiment, we intended to replicate our previous findings regarding the impairment of target search by comparing responses when the target was on one of the bars in the salient column (overlapping target) and on the other texture columns (non-overlapping target). More importantly, the overlapping probability was set on a chance level, as in Turatto and Galfano [2, 4]. To test the robustness of the effect, we allowed targets to appear in five possible locations in pre-specified 9 rows x 5 columns, which made it more difficult than having seven possible locations (a central row of only 7 columns), as in Jingling and Tseng [6]. Targets occurred at salient collinear distractor columns at the chance level (i.e., 20%).

The critical component of the design was that the locations of salient line and target were independently determined among the same five locations; salient line location was not informative about target location. According to Turatto and Galfano [2, 4], although the salient line was not relevant or informative to the target task, when our attention was captured by it, a target overlapping on it enjoyed the benefits of attention capture and, as such, required less time to be detected. However, according to our previous findings [6], an overlapping target turned out to be harder to be discriminate than was a non-overlapping target.

### Participants

Twenty-two undergraduate students at China Medical University were recruited and kept naïve about the goal of the study. Their time was compensated with 100 NTD or additional course credits. They had normal or corrected-to-normal vision, according to self-reports.

### Stimuli and Procedures

Observers saw 576 white element bars arranged in 21 rows x 27 columns against a dark background (Fig 1A) displayed on a 21” ViewSonic monitor. The luminance level was 120.05 cd/m^2^ for white and 0 cd/m^2^ for black. Each bar was .81° by .18° in the visual angle, with a viewing distance of 60 cm. One of the bars contained a tilt gap in the middle, and observers performed an orientation discrimination task to report which side this gap tilted toward (left or right). The target location varied from trial to trial, chosen among five columns (10th, 12th, 14th, 16th, and 18th) and nine rows (7th ~ 15th) by equal opportunity. One of the five columns was chosen as the distractor column; all element bars in this column were rotated by 90° and formed a salient, collinear structure. There was 1.04° of space between any two element bars. The distractor column was independently and randomly chosen, so only in one-fifth of the trial did the target overlap with the collinear column (namely, overlapping target). The search display stayed on the screen until the participants’ responses were received, and we encouraged our participants to respond as quickly as possible without sacrificing accuracy. Each participant completed 200 trials, which took approximately 15 minutes. The experiment was controlled with Psychtoolbox 2.54 in Matlab [34, 35].

## Results of Experiment 1

**Fig 3:**
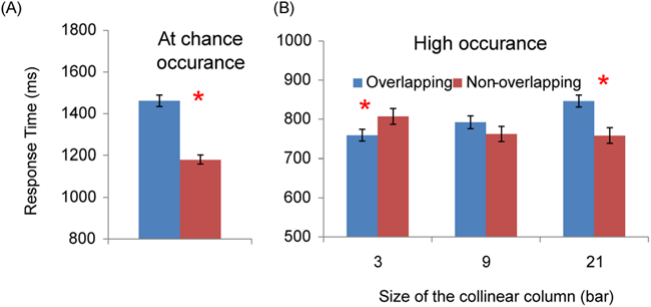
Results of chance occurrence in Experiment 1 (A), and high occurrence in Experiment 2 (B). Red columns are data for non-overlapping targets, while blue columns are data for overlapping targets. The asterisk indicates significant differences (*p* < .05) between conditions.

Only correct responses within two standard deviations above the grand mean of response times (RT) were kept for analysis. Thus, 4.41% of trials were discarded under this criterion. The selected data are shown in Fig 3A. We replicated the RT search impairment of overlapping targets [*t* (21) = 6.56, *p* < .001, RT_overlapping_ = 1461.74 ms, RT_non-overlapping_ = 1180.97 ms] as well as accuracy impairment (*t* (21) = 3.41, *p* < .001, Accuracy_overlapping_ = 92.05%, Accuracy_non-overlapping_ = 95.88%] in Jingling and Tseng’s study [6]. This is the opposite of what Turatto and Galfano [2, 4] discovered. Because accuracy data and RTs are known to diverge from a normal distribution, we did log-transformation of the data uphold the normality assumption in ANOVA testing. The analysis results after transformation were the same as that with raw data in all 4 experiments. In the text we follow the convention to report the analysis results with raw data only.

### Discussion of Experiment 1

We replicated the robust main finding in our previous observations [6] that a target overlapped with a salient distractor took observers longer to discriminate its orientation than when it overlapped with the background. In our search display, a collinear structure that was task irrelevant (i.e., its presence and location were uninformative to target orientation report) slowed down observers’ responses.

Our next experiment evaluated the modulation from probability distribution and top-down suppression. In Experiment 1, the target occurred on the collinear column only by chance (i.e., 20%), and the accumulation probability for all non-collinear columns was four times greater than it was for the collinear column. It is possible that observers, based on this probability information, strategically suppressed the space occupied by the collinear column. In Experiment 2, we increased the probability of target occurrence on the collinear column to 60%. The search disadvantage from the overlapped target should have decreased or reversed if observers were able to optimize their search behavior based on the statistical information.

## Experiment 2: High Occurrence of Overlapping Target

### Participants

Sixteen undergraduate students in China Medical University were recruited for Experiment 2. They had normal or corrected-to-normal vision and did not participate in the previous experiment.

### Stimuli and Procedures

All details were identical to those in Experiment 1 except that: (a) the target overlapped with the collinear column in 57.14% of the trials and appeared evenly at six other non-collinear columns in 42.86% of the trials (~6% each). This made the collinear column a predictor to target location; and (b) the collinear column length varied among 3, 9, and 21 bars (Fig 2B). According to Jingling and Tseng [6], the collinear column masked the target only when the length was long enough (e.g., 9 or 21 bars). Further, the target was always fixed at a central (11th) row instead of varying among seven possible rows, as in Experiment 1; thus, the task was relatively easier. Each participant completed 294 trials.

### Results of Experiment 2

We discarded the inaccurate trials and those with RT two standard deviations above the group mean; 2.38% of the trials were excluded. Results, shown in Fig 3B, were submitted to a two (collinear length 3, 9, or 21 bars) by two (target overlapping or non-overlapping with the collinear distractor) repeated ANOVA. We found that RT was longer when the distractor column length was long: *F* (2, 15) = 4.50, *MSE* = 1218.44, *p* < .05. Meanwhile, we found a significant interaction between distractor length and target type: *F* (2, 30) = 28.85, *MSE* = 1298.56, *p* < .0001. Simple main effect analysis on this interaction showed that overlapping targets (758.98 ms) were discriminated *faster* than non-overlapping targets (807.19 ms) when the distractor column was at size 3, *F* (1, 45) = 7.29, *MSE* = 2552.10, *p* < .01. Yet, the effect reversed when the distractor column was at size 21 (overlapping targets 846.33 ms vs. non-overlapping targets 758.15 ms, *F* (1, 45) = 24.37, *MSE* = 2552.10, *p* < .0001). Therefore, even if the collinear column predicted the target location, it still impaired target discrimination when the column was long enough. The ANOVA analysis on accuracy data did not find any significant difference, arguing against possible speed—accuracy trade-off.

### Discussion of Experiment 2

In Experiment 2, the distractor column was predicative to the target location, and observers could use this informative cue to enhance their search performance by directing their attention to this salient structure in the search display. However, observers continued to suffer at trials where the target overlapped with the distractor column when the column was long. The results speak against the low-probability hypothesis, which suggests that the visual search impairment in Experiment 1 was due to statistical information.

Muller et al. [8] systematically varied the portion of a task-irrelevant singleton distractor to appear in a search display where observers looked for a shape-defined target. They found that the interference from the infrequent singleton distractor was significantly bigger than frequent ones, implying that observers did use the probabilistic information of distractor presence to customize their search strategy. In our design, the distractor was always present, but its predictability about target location varied. At higher occurrence condition (Exp 2), observers actually benefited from directing their attention to the distractor column, as the target fell on this column for 60% of the trails. However, this was only visible from observers’ response time when the distractor was short (3-bar condition), which is not applicable to long-distractor conditions (9-, 21-bar). The characteristic difference between the short and long collinear column suggested that regardless of the probability of the target occurrence overlapping with the distractor column, a penalty seemed to be imposed exclusively on the long collinear structure. Can this be owing to observers’ limited access to this information due to factors such as unawareness of the statistical distribution of the target location? Statistical learning has been reported in tasks embedding more implicit probabilistic information, even when observers were unaware of it [18–23]. More plausibly, we speculate that collinear impairment may occur at a perceptual level that is not heavily modulated by event probable occurrence.

## Experiment 3: Luminance Discrimination Target Task

In this experiment, we tested whether the search impairment effect for a local target overlapping with a salient collinear column occurred only when a distractor property (e.g., continuous collinear structure) mismatched target task nature (e.g., a breakdown of continuity). In this experiment, we retained the same search display but re-designed the target task to be a luminance discrimination task so that they did not conflict with the continuous distractor. If task set is important, than search impairment effect is not expected in this experiment.

### Participants

Twelve undergraduates in China Medical University were recruited and compensated with additional course credits or 100 NTD. They claimed to have normal or corrected-to-normal vision. They were not told about the goal of this study in advance and did not participate in the previous experiment.

### Stimuli and Procedures

**Fig 4:**
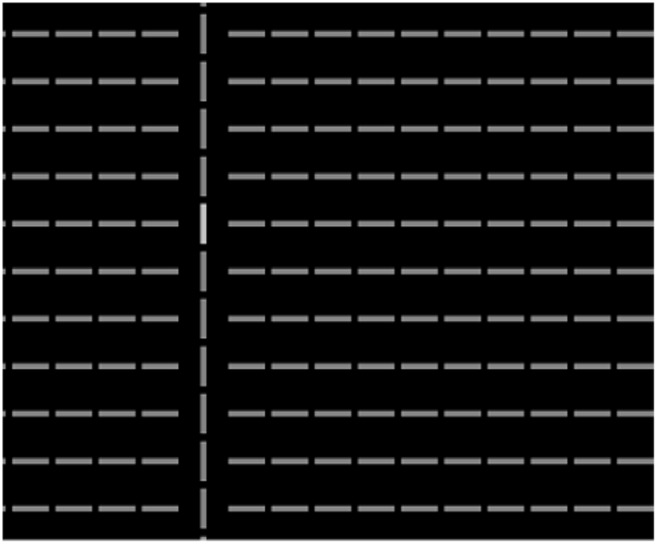
Part of the search display used in this experiment. The target is a brighter bar and overlaps with the salient collinear column in this example.

The experiment was similar to that in Jingling and Tseng [6]. Fig 4 shows part of the search display. The whole search display was filled with 21 rows and 27 columns of gray bars (128 of 256 luminance level, 41.9 cd/m^2^) on black background (0 cd/m^2^). These bars were all horizontal, except for one odd-man-out distractor column of vertical bars. The distractor column could have three or 21 bars in vertical, which is called *distractor size* hereafter. Participants discriminated whether the target was a brighter (192 over 256 luminance level, 82.4 cd/m^2^) or a darker (64 over 256 luminance level, 17.5 cd/m^2^) bar in each trial by pressing corresponding keys. There were seven possible columns (8th, 10th, 12th, 14th, 16th, 18th, and 20th) of the total 27 columns that could present the target or the column of the distractor. The targets’ vertical position was fixed at the central (11th) row. Positions of the target and the distractor were manipulated orthogonally, making the distractor and the target spatially irrelevant. Each participant completed 392 trials after 10 practice trials.

### Results of Experiment 3

**Fig 5:**
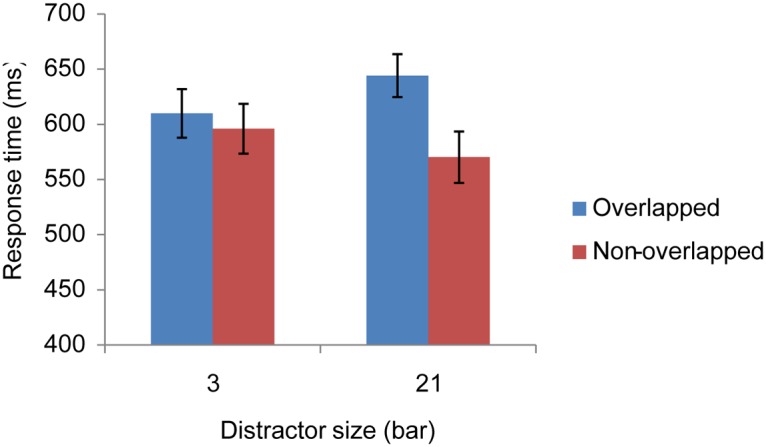
Results of Experiment 3. The error bars are the standard error of the mean.

We excluded incorrect trials (4.31%) and those trials (2.87%) which took longer than two standard deviations of the grand mean. The selected RTs were then submitted to a two-way repeated ANOVA, with target type (overlapped or non-overlapped) and distractor size (3 or 21 bars) as factors. Fig 5 shows the results of the selected RT. The main effect of target type was found, *F* (1, 11) = 26.43, *MSE* = 873.11, *p* < .001. That is, responses were longer for overlapping targets (626.89 ms) than they were for non-overlapping targets (583.06 ms). Also, the two-way interaction was significant, *F* (1, 11) = 18.60, *MSE* = 580.96, *p* < .001. As shown in Fig 5, responses for overlapping targets were slower than those for non-overlapping targets when the distractor column was long, *F* (1, 22) = 45.02, *MSE* = 727.03, *p* < .0001, but not when the distractor column was short, *p* = .22. This result replicated that in Jingling and Tseng [6], suggesting that a similar search impairment effect can be observed with a different task set.

The overall accuracy was 92.84%. Accuracy data were also submitted to a two-way repeated ANOVA, and no effect was significant. There was no evidence of a speed—accuracy trade-off.

### Discussion of Experiment 3

The search disadvantage for overlapping targets persisted when the target task was replaced with a luminance discrimination task. In the contingent capture hypothesis, visual features that are irrelevant to the behavioral goals are filtered out and do not cause involuntary attentional shifts [10]. Our result shows that attentional control setting is not a major factor that drives this effect. Rather, it is the composition of the display—particularly, the continuous collinearity—that slows down target processing when it is spatially overlapped with the collinear structure. The target processing, regardless of orientation judgment or luminance discrimination, was interfered with. The mental set to prepare attention ready for a particular dimension is not the cause of this phenomenon.

## Experiment 4: Visual Search Practice

To understand whether the search impairment effect was diminished after intense practice, we repeated the search task for 10 days. If the search impairment were under top-down control or a result of search strategy, a reduction of the impairment along experience would be expected. If, however, this effect were mainly induced by stimulus properties (e.g., salience or collinear grouping), practice should not alter the amount of such effect.

### Participants

Eight graduate students in China Medical University joined to complete one session a day for 10 consecutive days. They were naïve about the goal of this study and had not had experience on similar search tasks before. They had normal or corrected-to-normal vision. They received 1000 NTD for a reward.

### Stimuli and Procedures

Two factors were designed in this experiment: distractor size and target overlapping or not. The experiment was identical to Experiment 4 in Jingling and Tseng [6]: each session contained 294 trials, with seven possible target (and distractor) locations X 3 distractor size. Ten practice trials were completed before each session. Participants discriminated the target tilt as soon as possible. These four participants completed 10 sessions in 10 days.

### Results of Experiment 4

**Fig 6:**
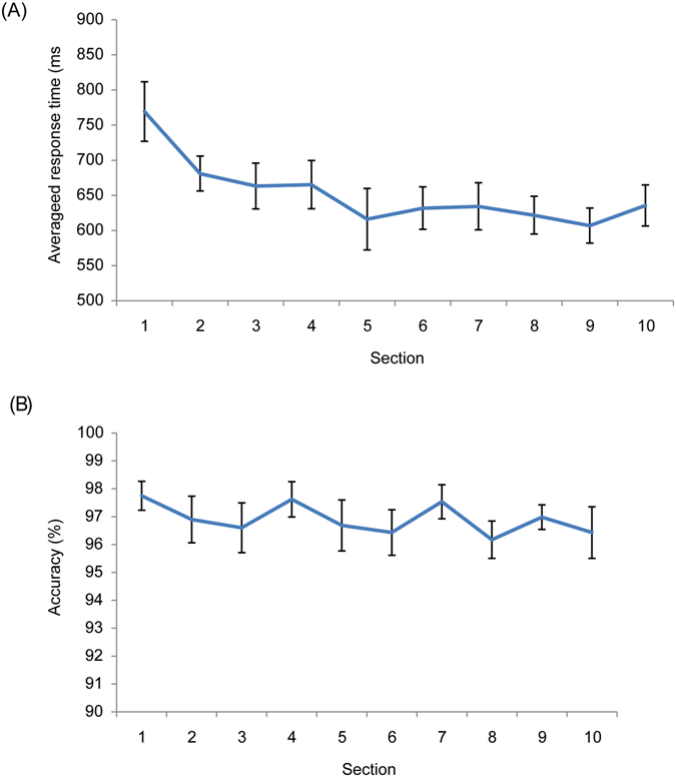
Overall responses across 10 sections. The upper panel is data of response times, while the lower panel is accuracy. The error bars are the standard error of the mean.

Inaccurate trials (3.62%) or the trials with RT exceeding two standard deviations of the individual mean (3.70%) were excluded. As shown in Fig 6 (upper panel), we found that overall RT decreased with practice, from 769.16 ms for the first section reduced to 635.51 ms for the last section. However, significant improvement was found only for the first four sections, *F* (9, 63) = 6.36, *MSE* = 8413.12*p* < .01, while the RTs hardly improved in the remaining sections. The overall accuracy did not significantly vary across sections, *p*s > .05.

**Fig 7:**
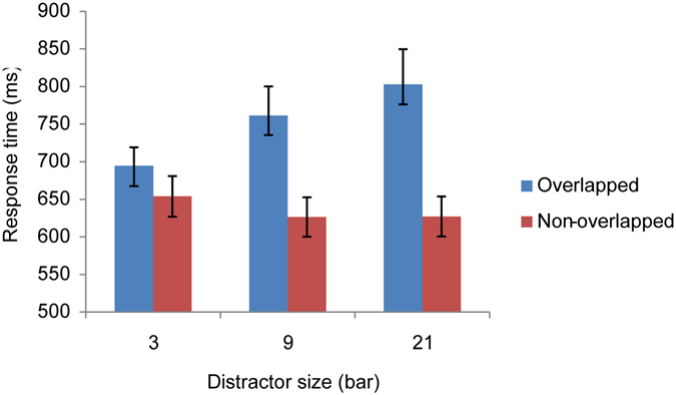
RTs of each condition for the four participants. The error bars are the standard error of the mean.

The general responses across all observers and sections (Fig 7) replicated the findings in [6]. Results collapsed across observers were submitted to a 10 (sections) by 3 (distractor size, 3, 9, or 21 bars) by 2 (target type, overlapping or non-overlapping) repeated-measure ANOVA. We observed the signature patterns here also: significantly higher RTs and lower accuracy for overlapping targets (694.58 ms, 87.92%) than for non-overlapping targets (612.53 ms, 94.14%), *F* (1, 7) = 160.97 and 30.25, *MSE* = 5506.14 and .015, *p*s < .001, respectively. Significant interaction between distractor size and target type, *F* (2, 14) = 41.18 and 6.36, *MSE* = 1530.83 and .014, *p*s < .001, respectively. Overlapping target detection was hurt only when the distractor size was 9-bar or 21-bar; *F* (1, 21) = 135.99 and 194.40, respectively; *MSE* = 2855.93, *p*s < .001 for RT; *F* (1, 21) = 14.19 and 29.50, respectively; and *MSE* = .015, with *p*s < .001 for accuracy.

**Fig 8:**
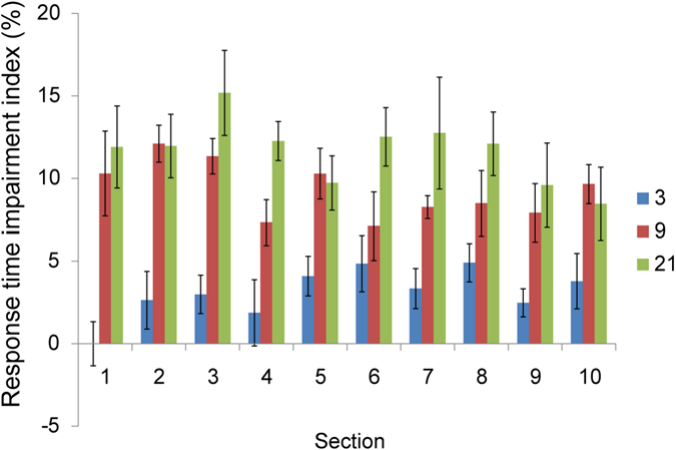
Search impairment index on RT in each session. The error bar is the standard error of the mean.

To understand how participant’s search impairment varied with sections, we normalized observers’ search impairment by introducing a search impairment index (SI), defined by the difference between RT of trials with overlapping targets and non-overlapping targets, divided by the mean RT [37]. A positive value refers to search impairment, and negative value refers to search advantage of overlapping targets. Fig 8 shows SI of three conditions in each day. As revealed by the 3-waya ANOVA, these effects did not vary with sessions, suggesting that practice did not reduce the size of search impairment in overlapping targets.

### Discussion of Experiment 4

With sufficient practice, observers continued to suffer from having slower reaction times and higher error rates when the searched-for targets were on the collinear distractor column if the collinear length were long enough. The extensive practice in 10 days was not enough to let observers develop strategies to facilitate local target orientation processing hampered by the background contextual organization.

Kelly and Yantis [12] reported observers’ capacity to learn to ignore task-irrelevant distractors. In their design, an abrupt-onset distractor showed up in the screen corner 100ms before a central 5x5 array appeared. The target task required observers’ judgment whether more red or green dots were presented in the central array. At first, observers’ accuracy was reduced due to the distractor’s presence, but it recovered to the same level as in the no-distractor condition at the end of 1,000 practice trials. Also, observers’ improved ability to filter out task-irrelevant information was limited to the trained spatial locations and distractor types. However, if the distractor-to-learn was heterogeneous enough (i.e., a set of 520 distinct images), then the learning effect transferred across location and distractor types. This shows observers are flexible and can enable learning at multiple facets from very locally-limited information to a more global information efficiency enhancement. Our observers did not benefit practice, and there were several notable differences between the two studies worth mentioning.

First, the distractor and target in Kelly and Yantis [12] were separated in space and time (100 ms), while in our case, the target was always on one of the element distractors. The spatial-temporal characteristics of the target-distractor may be a limiting factor for learning effect, which has not been studied much. The gradual reduced response time from the first few sessions was likely from faster target detection, key press, or decision making after familiarity of the task.

Secondly, feedback was provided with a reward matrix in Kelly and Yantis [12], which could serve as a strong guidance for search strategy optimalization. In our case, observers were instructed to respond as fast as possible without sacrificing accuracy. No feedback was provided in our study, and our observers’ accuracy was at a level (92.69%) comparable to other similar tasks requiring observers’ fast response. Despite these differences, the clear signature difference between overlapping and non-overlapping conditions throughout all 10 sessions disagrees with the hypothesis that this impairment is from sub-optimal search strategy due to lack of exposure. More plausibly, the display property and stimuli spatial arrangement are the fundamental limits for observers to discriminate the local orientation.

## General Discussion

In this study, we tested three top-down hypotheses about the origin of the visual search impairment generated by the presence of collinear task-irrelevant distractors in a search display. Our results did not support the view that collinear distractors were suppressed due to their low probabilistic occurrence of overlapping with the primary target, their attentional control setting in specific tasks, or lack of learning regarding search strategy development.

Ecologically, both selective attention and perceptual grouping are both processes that reduce our information processing load. The former directs our mental resources to the spatial areas or features that deserve more processing. The latter enhances our efficiency by quickly sorting properties likely to be possessed by the same surface or object into one grouped unit. It is not surprising that these two processes serving the same purpose interact at some point in our visual processing, but the potential neural platform and the cause of this interaction (whether it is stimulus-driven or modulated by cognition) is still largely unknown.

Contextual background information, even task-irrelevant, was influential in local target tasks. Driver et al. [38] reported that target luminance change detection was modulated by the background context arrangement and that the detection was easier when the target was seen as the foreground. Kimchi and colleagues [39] found that task-irrelevant element configuration facilitated target color identification when the target located within an object was formed by Gestalt factors such as collinearity, closure, and symmetry. An opposite cost effect was observed when the target was located outside of such object. Similar effect was observed when the target task was replaced by a vernier judgment, suggesting an attentional facilitation from a perceptual object induced by collinear/closure perceptual grouping principles [40]. In our case, the collinear grouping of the distractor is most intuitively taken as a popped-out distinctive structure (Fig 1). However, it could just as well be taken as a divider that separates the search display into two large areas, thereby globally seen as a negligible part to which attention is rarely directed. The “distinctive structure” or “divider” view is a figure—ground perspective that can be task-defined, and the interpretation can be different or opposite in different contexts. If the collinear column in a search display is defined as a divider (i.e., ground) rather than an object, it may lead to low salience and, thus, slower response time. Indeed our eye movement study suggested that overlapping targets were perceptually less salient than non-overlapping targets [6]. Because the task-dependent and context-dependent nature in figure/ground interpretation, it is beyond the bottom-up simple feature contrast computation. It requires some thought to incorporate it into a visual search model.

Although the interpretation of figure/ground suggests the modulation from top-down influence (e.g., task—demand), it was not supported by available models and empirical results in the case of collinear search impairment. The V1 saliency model proposed by Li [41] and Zhaoping [42] is the most relevant architecture that concerns collinear facilitation in addition to other basic visual features to predict how attention is deployed and attenuated. In our search display, all the component bars in the collinear column were orthogonal to their neighboring bars, thus yielding the maximum local contrast salience. According to the V1 saliency model, the integrated result from local-contrast salience and collinear grouping will determine the V1 neuronal activation and subsequent search performance. In other words, whether oriented bars aligned to each other directly contribute to perceptual salience. As demonstrated in Jingling and Zhaoping [43], collinearly grouped texture boundary created higher salience than un-grouped boundary. Beyond the local feature contrast computation, any structure grouped by continuity will require a second-order computation that determines the strength of this structure in relation to other regions. The size effect observed here can be explained by the competition between the local contrast salience computation and the second-order salience computation based on perceptual grouping laws. At one end, when the collinear grouping is weak (i.e., three bars), the first-order salience computation dominates; therefore, conventional attention capture facilitation is observed when the target overlaps with the salient distractor. At the other end, when the perceptual grouping strength is increased, the second-order computation dominates attentional selection in a visual search. The above-mentioned computation is described to automatically (i.e., bottom-up) take place at V1.

The V1 saliency model received support from an empirical study that utilized the collinear impairment size effect and binocular fusion to explore interaction neural sites for selective attention and perceptual collinearity. Chow, Jingling, and Tseng [37] separated the task-irrelevant collinear distractor column into parts and presented them to individual eyes with a stereoscope. Each eye only saw a small part too short to elicit search impairment, but when binocularly fused, the combined structure exceeds the length to slow down the target search. The result indicated that monocular information dictated the search performance, implying the origin of eye information was available even when observers were not unaware of it. This puts V1, the cortical area containing the most monocular cells [44–45], a highly probable candidate for the collinear grouping to interact with selective attention.

Another promising direction is the insight derived from the perceptual grouping model proposed by Roelfsema and Houtkamp [46]. In this model, perceptual grouping involves two level of processes (i.e. base-grouping and incremental grouping). During base-grouping, individual neurons are activated based on their intrinsic preferences toward selective visual features such as orientation. Later a feedforward and recurrent processing bring in environmental information and our attentive selection. This multi-layered computation of grouping offers an architecture of grouping principles that “collinearity” becomes a property not exclusively processed at a single stage. In other words, collinearity can evoke strong activation in base-grouping due to neurons’ orientation consistency, as well as a strong activation from recurrent lateral connections that might involve attention. Our experiments have suggested that learning, probability, and attentional control setting on task set all have little effect to overcome the collinear impairment. It may imply that it was the base group principles that matter most.

Taken together, the converging evidence suggests that collinear search impairment is a stimulus-driven interference from perceptual grouping. Without observers’ awareness, the origin of the eye is reserved in determining the attentional search behavior. This effect is not easily maneuvered by cognitive modulations including statistical regularity information of target presence and location, attentional set control, or extensive learning. It is still an open question whether all grouping principles facilitate visual search and constrain selective attention in the same way. This will be a direction for future studies.

## Conclusions

In summary, most models of visual search have extensively considered the dynamics of how salience information is formulated by various types of bottom-up attributes with stimuli and distractors that are similar in size. Similar efforts have also been invested to explore the role of top-down control. Our study signifies the need for additional consideration of mid-level computation, such as perceptual grouping and its role in guiding visual search. Our observations also lead us to suggest that (1) perceptual grouping interacts with visual attention in a distinct way from bottom-up salience defined by feature contrast, and therefore should be considered separately; and (2) collinear grouping is less modulated by probability information or the top-down strategy developed upon it. These two features should be incorporated into a future extension of visual selection models.

## References

[1] Theeuwes J. Top—down and bottom—up control of visual selection. Acta psychol. 2010; 135(2): 77–99. 10.1016/j.actpsy.2010.02.006 20507828

[2] Turatto M, Galfano G. Color, form and luminance capture attention in visual search. Vision Res. 2000; 40, 1639–1643. 10814751 10.1016/s0042-6989(00)00061-4

[3] Jonides J, Yantis S. Uniqueness of abrupt visual onset in capturing attention. Percept Psychophys. 1988; 43, 346–354. 3362663 10.3758/bf03208805

[4] Turatto M, Galfano G. Attentional capture by color without any relevant attentional set. Percept Psychophys. 2001; 63, 286–297. 11281103 10.3758/bf03194469

[5] Yantis S, Jonides J. Abrupt visual onsets and selective attention: evidence from visual search. J Exp Psychol Human. 1984; 10, 601–621. 6238122 10.1037//0096-1523.10.5.601

[6] Jingling L, Tseng CH. Collinearity impairs local element visual search. J Exp Psychol Human. 2013; 39, 156–167. 10.1037/a0027325 22329767

[7] Geyer T, Müller HJ, Krummenacher J. Expectancies modulate attentional capture by salient color singletons. Vision Res. 2008; 48, 1315–1326. 10.1016/j.visres.2008.02.006 18407311

[8] Müller HJ, Geyer T, Zehetleitner M, Krummenacher J. Attentional capture by salient color singleton distractors is modulated by top-down dimensional set. J Exp Psychol Human. 2009; 35, 1–16. 10.1037/0096-1523.35.1.1 19170466

[9] Sayim B, Grubert A, Herzog MH, Krummenacher J. Display probability modulates attentional capture by onset distractors. J Vision. 2010; 103, 1–8. 10.1167/10.3.10 20377287

[10] Folk CL, Remington RW, Johnston JC. Involuntary covert orienting is contingent on attentional control settings. J Exp Psychol Human. 1992; 18, 1030–1044. 1431742

[11] Folk CL, Remington RW. Selectivity in distraction by irrelevant featural singletons: Evidence for two forms of attentional capture. J Exp Psychol Human.1998; 24, 847–858. 9627420 10.1037//0096-1523.24.3.847

[12] Kelley T, Yantis S. Learning to attend: Effects of practice on information selection. J Vision. 2009; 9, 1–18. 10.1167/9.7.16 PMC3124869 19761331

[13] Cohen A, Magen H. Intra- and cross-dimensional visual search for single-feature targets. Percept Psychophys. 1999; 61, 291–307. 10089762 10.3758/bf03206889

[14] Mortier K, Theeuwes J, Starreveld P. Response Selection Modulates Visual Search Within and Across Dimensions. J Exp Psychol Human. 2005; 31, 542–557. 15982130 10.1037/0096-1523.31.3.542

[15] Theeuwes J. Cross-dimensional perceptual selectivity. Percept. Psychophys. 1991; 50, 184–193. 1945740 10.3758/bf03212219

[16] Theeuwes J. Perceptual selectivity for color and form. Percept. Psychophys. 1992; 51, 599–606. 1620571 10.3758/bf03211656

[17] Theeuwes J, Reimann B, Mortier K. Visual search for featural singletons: No top-down modulation, only bottom-up priming. Vis Cogn.2006; 14, 466–489.

[18] Chun MM, Jiang Y. Contextual cueing: implicit learning and memory of visual context guides spatial attention. Cognitive Psychol. 1998; 36, 28–71. 9679076 10.1006/cogp.1998.0681

[19] Fiser J, Aslin RN. Unsupervised statistical learning of higher-order spatial structures from visual scenes. Psychol Sci. 2001; 12, 499–504. 11760138 10.1111/1467-9280.00392

[20] Fiser J, Aslin RN. Statistical learning of higher order temporal structure from visual shape sequences. J Exp Psychol Learn. 2002; 28, 458–467. 12018498 10.1037//0278-7393.28.3.458

[21] Geng JJ, Behrmann M. Probability cuing of target location facilitates visual search implicitly in normal participants and patients with hemispatial neglect. Psychol Sci. 2002; 13, 520–525. 12430835 10.1111/1467-9280.00491

[22] Geng JJ, Behrmann M. Spatial probability as an attentional cue in visual search. Percept Psychophys. 2005; 67, 1252–1268. 16502846 10.3758/bf03193557

[23] Turk-Browne NB, Jungé JA, Scholl BJ. The automaticity of visual statistical learning. J Exp Psychol Gen. 2005; 234, 552–564. 10.1037/0096-3445.134.4.552 16316291

[24] Farrell S, Ludwig CJ, Ellis LA, Gilchrist ID. Influence of environmental statistics on inhibition of saccadic return. P Natl Acad Sci USA. 2009; 107, 929–934. 10.1073/pnas.0906845107 PMC2818969 20080778

[25] Liu CL, Chiau HY, Tseng P, Hung DL, Tzeng OJL, Muggleton NG, et al The antisaccade cost is modulated by contextual experience of location probability. J Neurophysiol. 2010; 103, 1438–1447. 10.1152/jn.00815.2009 20032240 PMC2887634

[26] Folk CL, Annett S. Do locally defined feature discontinuities capture attention? Percept Psychophys. 1994; 56, 277–287. 7971128 10.3758/bf03209762

[27] Bacon WF, Egeth HE. Overriding stimulus-driven attentional capture. Percept Psychophys. 1994; 55, 485–496. 8008550 10.3758/bf03205306

[28] Gibson BS, Kelsey EM. Stimulus-driven attentional capture is contingent on attentional set for displaywide visual features. J Exp Psychol Human. 1998; 24, 699–706. 9627409 10.1037//0096-1523.24.3.699

[29] Jingling L, Yeh SL. New objects do not capture attention without a setting: Evidence from inattentional blindness. Vis Cogn. 2007; 15, 661–684.

[30] Liu L, Kuyk T, Fuhr P. Visual search training in subjects with severe to profound low vision. Vision Res.2007; 47, 2627–2636. 17707452 10.1016/j.visres.2007.07.001

[31] Sireteanu R, Rettenbach R. Perceptual learning in visual search: Fast, enduring, but non-specific. Vision Res.1995; 35, 2037–2043. 7660607 10.1016/0042-6989(94)00295-w

[32] Sireteanu R, Rettenbach R. Perceptual learning in visual search generalizes over tasks, locations, and eyes Vision Res. 2000; 40, 2925–2949. 11000393 10.1016/s0042-6989(00)00145-0

[33] Hess RF, Dakin SC, Field DJ. The role of “contrast enhancement” in the detection and appearance of visual contours. Vision Res. 1998; 38, 783–787. 9624429 10.1016/s0042-6989(97)00333-7

[34] Brainard DH. The psychophysics toolbox. Spatial Vision. 1997; 10, 433–436. 9176952

[35] Pelli DG. The VideoToolbox software for visual psychophysics: transforming numbers into movies. Spatial Vision. 1997; 10, 437–442. 9176953

[36] Chun MM, Jiang Y. Implicit, long-term spatial contextual memory. J Exp Psychol Learn. 2003; 29, 224–234. 12696811 10.1037/0278-7393.29.2.224

[37] Chow HM, Jingling L, Tseng CH. Collinear integration affects visual search at V1. J Vision. 2013; 13, 1–20. 10.1167/13.10.24 23988390

[38] Driver J, Davis G, Russell C, Turatto M, Freeman E. Segmentation, attention and phenomenal visual objects. Cogn. 2001; 80, 61–95. 10.1016/s0010-0277(00)00151-7 11245840

[39] Kimchi R, Yeshurun Y, Cohen-Savransky A. Automatic, stimulus-driven attentional capture by objecthood. Psychon B Rev. 2007; 14, 166–172. 17546748 10.3758/bf03194045

[40] Yeshurun Y, Kimchi R, Sha’shoua G, Carmel T. Perceptual objects capture attention. Vision Res. 2009; 49, 1329–1335. 10.1016/j.visres.2008.01.014 18299141

[41] Li Z. A saliency map in primary visual cortex. Trends Cogn Sci. 2002; 6, 9–16. 11849610 10.1016/s1364-6613(00)01817-9

[42] Zhaoping L. The primary visual cortex creates a bottom-up saliency map In: Itti L, Rees G, Tsotsos JK, editors. Neurobiology of attention. Elsevier: Amsterdam; 2005 pp. 570–575.

[43] Jingling L, Zhaoping L. Change detection is easier at texture border bars when they are parallel to the border: Evidence for V1 mechanisms of bottom-up salience. Perception. 2008; 37(2), 197–206. 18456924 10.1068/p5829

[44] Hubel DH, Livingstone MS. Segregation of form, color, and stereopsis in primate area 18. J Neurosci. 1987; 7, 3378–3415. 2824714 10.1523/JNEUROSCI.07-11-03378.1987 PMC6569042

[45] Hubel DH, Wiesel TN. Receptive fields and functional architecture of monkey striate cortex. J Physiol. 1968; 195, 215–243. 4966457 10.1113/jphysiol.1968.sp008455 PMC1557912

[46] Roelfsema RPR, Houtkamp R. Incremental grouping of image elements in vision. Attention Perception Psychophys. 2011; 73(8), 2542–2572. 10.3758/s13414-011-0200-0 21901573 PMC3222807

